# The Efficacy of Dandelion Root Extract in Inducing Apoptosis in Drug-Resistant Human Melanoma Cells

**DOI:** 10.1155/2011/129045

**Published:** 2010-12-30

**Authors:** S. J. Chatterjee, P. Ovadje, M. Mousa, C. Hamm, S. Pandey

**Affiliations:** ^1^Department of Chemistry and Biochemistry, University of Windsor, 401 Sunset Avenue, Windsor, ON, Canada N9B 3P4; ^2^Windsor Regional Cancer Centre, 2220 Kildare Road, Windsor, ON, Canada N8W 2X3

## Abstract

Notoriously chemoresistant melanoma has become the most prevalent form of cancer for the 25–29 North American age demographic. Standard treatment after early detection involves surgical excision (recurrence is possible), and metastatic melanoma is refractory to immuno-, radio-, and most harmful chemotherapies. Various natural compounds have shown efficacy in killing different cancers, albeit not always specifically. In this study, we show that dandelion root extract (DRE) specifically and effectively induces apoptosis in human melanoma cells without inducing toxicity in noncancerous cells. Characteristic apoptotic morphology of nuclear condensation and phosphatidylserine flipping to the outer leaflet of the plasma membrane of A375 human melanoma cells was observed within 48 hours. DRE-induced apoptosis activates caspase-8 in A375 cells early on, demonstrating employment of an extrinsic apoptotic pathway to kill A375 cells. Reactive Oxygen Species (ROS) generated from DRE-treated isolated mitochondria indicates that natural compounds in DRE can also directly target mitochondria. Interestingly, the relatively resistant G361 human melanoma cell line responded to DRE when combined with the metabolism interfering antitype II diabetic drug metformin. Therefore, treatment with this common, yet potent extract of natural compounds has proven novel in specifically inducing apoptosis in chemoresistant melanoma, without toxicity to healthy cells.

## 1. Introduction

Melanoma skin cancer is among one of the leading cancers targeting adolescents and young adults in the North America. Melanoma is notoriously chemo-resistant and the modes of treatment for melanoma are very limited, relying mainly on surgical excision of the primary site during early detection, and whatever limited chemotherapy and immunotherapy for metastasized melanoma that is available. However, these therapies have limited success and incur side effects [1]. 

*Taraxacum Officinale* is most commonly known as dandelion. Regarded as a regular garden weed, this detoxifying herb has long been used in traditional Chinese medicine, for ailments ranging from digestive disorders to complex disorders such as uterine, breast and lung tumours [2]. Traditional Middle Eastern remedies require dandelions for spleen and liver ailments, while Native Americans have harnessed their properties to cure indigestion, heartburn, and kidney disease [2, 3]. Dandelion plants were (*Taraxacum officinale* Weber ex Wiggers) were used by East Indians in the 16 century as a hepatic stimulant, diuretic, for liver disorders, and most interestingly, for chronic skin diseases [4, 5]. These roots are a source of triterpenes and steroids [4, 6]. Hata et al. found that upon screening a variety of compounds from wild plants, *Taraxacum Officinale* was an effective inducer of differentiation in mouse melanoma cells. Furthermore, this group found that one constituent of Chinese dandelion, Lupeol-a triterpene, up-regulated melanogenesis and decreased cell proliferation in mouse melanoma [7]. This triterpene is regarded as cytostatic and not cytotoxic. In another study, skin tumours were promoted *in vivo* in mice through a two-stage chemical carcinogenesis and treated with water and methanol extracts of *Taraxacum japonicum*. This showed inhibition of tumour initiation and promotion at both carcinogenesis stages, and it was concluded that Taraxacum, and more specifically taraxasterol (a triterpenoid), is a worthy chemopreventative agent [8, 9]. 

More recently, Jeon et al. have shown that ethanolic *Taraxacum Officinale* extracts and derivative forms thereof reduce levels of reactive oxygen species (ROS) and nitric oxide production (NO) and inhibit COX-2 expression or its antioxidant activity, thus making dandelion extracts not only anti-carcinogenic, but anti-inflammatory, antiangiogenic and also antinociceptive [10]. Dandelion flower extracts (DFE) were tested on RAW264.7 cells (mouse macrophages) and exhibited inhibition of NO production in these noncancerous cells. The inhibition of reactive nitrogen species (RNS) as well as ROS by DFE was attributed to its phenolic components [11]. Sigstedt et al. tested aqueous DFE, dandelion leaf extract (DLE), and dandelion root extract (DRE), from *Taraxacum Officinale*, on a variant MCF-7 breast cancer cell line (MCF-7/AZ) and LNCaP prostate cancer line. While DLE inhibited cell proliferation in MCF-7/AZ, DFE and DRE did not. However, DRE and DLE blocked invasion of MCF-7/AZ and LNCaP cells (into collagen type I), respectively. Inhibition of cell invasion was corroborated by reduced matrix metalloproteinase activity of MMP-2 and -9, as well as reduced phosphorylation levels of src and FAK [2]. *Taraxacum Officinale *extract was used to treat Hep G2 human hepatoma cells and was found to reduce cell viability and induce cytotoxicity through interleukin-*α* and TNF-*α* [12]. Regardless of the valuable traditional knowledge of Taraxacum antitumor activity, there has been inadequate biochemical research to apply this knowledge to cancer cell lines and especially chemo-resistant melanoma. 

Population studies have highlighted the correlation between diabetic patients taking Metformin and a reduced incidence of cancer and lower rates of mortality. Jiralerspong et al. had clinically proven this by determining that diabetics with breast cancer taking Metformin had a higher pathological complete response than diabetic breast cancer patients not taking the drug [13]. Normally, healthy cells express adenosine monophosphate-activated protein kinase (AMPK), which is directly downstream of the tumour suppressor LKB1 and is usually activated as a reaction to cellular energy stress. Metformin can also activate AMPK, leading to suppression of the ATP consuming pathways (e.g., gluconeogenesis) and activation of the ATP generating pathways (e.g., glycolysis). Though the mechanisms for metformin treatment of cancer is not clearly understood; it is clear that cancer cell energy metabolism differs from that of normal cells, and this difference makes tumourigenic cells vulnerable to metabolism-interfering drugs like metformin [14]. 

In this study, we have investigated the effect of dandelion root extract on human melanoma cell lines *in vitro*. For melanoma, a very aggressive, chemo-resistant form of skin cancer, we have shown that DRE has been very effective in inducing apoptosis. We have also seen that the extract targets the mitochondria, generating reactive oxygen species. It is possible that the compounds in the extract work through the extrinsic apoptotic pathway, as is indicated by elevated levels of early caspase-8 activation. We have also observed that drug-resistant melanoma cells could be made more sensitive to DRE treatment by the metabolism interfering drug, metformin. This is the first time that a study has been performed with metformin and human melanoma. Though it is unclear which components of DRE are active in successfully killing human melanoma cells, our work with Taraxacum DRE presents a novel, natural chemotherapeutic agent that may be extended to other chemo-resistant cancer lines.

## 2. Materials and Methods

### 2.1. Cell Culture

Human melanoma cell lines, A375 and G361, were purchased from ATCC (Manasas, VA, USA). A375 human melanoma cells were grown and cultured at 37°C and 5% CO_2_ in RPMI-1640 media containing L-glutamine and NaHCO_3_ (Sigma-Aldrich, Oakville, ON, Canada) completed with 10% Fetal Bovine Serum (FBS) (Sigma, Canada) and 10 *μ*g/mL gentamicin (Gibco, Canada). G361 human melanoma cells were cultured in McCoy's Medium 5a modified with L-glutamine (Gibco, Canada) in the same manner as the A375s. 

Human nucleated blood cells were purified from whole blood obtained from healthy volunteers as approved by the University of Windsor ethical committee, REB# 04-060. Whole blood (12 mL) was collected into a BD Vacutainer CPT Tube (Cell Preparation Tube) obtained from Becton Dickinson (Franklin Lakes, N.J.). The whole blood was spun down in a table-top low-speed centrifuge at 2900 × g for 30 min at 25°C. The red blood cells went through the polyester gel and the top layer containing mononuclear cells, platelets, and plasma was collected. These peripheral mononuclear blood cells (PBMCs) were incubated at 37°C, 5% CO_2_, and 95% humidity. Normal human Fibroblasts (NHFs) were purchased from the Coriell Institute for Medical Research, USA. NHFs were cultured in Earle's Minimum Essential Medium (Sigma-Aldrich, Oakville, ON, Canada) completed with 15% FBS, 2 mM L-glutamine, 10 *μ*g/mL gentamicin, vitamins, and essential and nonessential amino acids (Gibco, Canada).

### 2.2. Cell Treatment

Water extracts of dandelion root were prepared using variety of filtering stages, lyophilized, constituted to a particular stock concentration and sterilized. Washed roots were blended in water until macerated. The blended mass was then passed through a nylon mesh to filter out very large fibres. The filtrate was then centrifuged at 5000 g for 5 minutes at room temperature. The supernatant was then filtered through a Buchner funnel. The filtrate was then passed through a 0.45 micron filter then a 0.2 micron filter, before being lyophilized to a fine powder. The powder is then reconstituted with sterile 1X Phosphate Buffer Saline (PBS) to a particular stock concentration after which the DRE is further diluted upon cellular treatment in media. Cells (A375 and G361 human melanoma cells, and NHFs) were treated with DRE at a 60% confluence, at the indicated concentrations and time points. PBMCs were grown to 70% confluence and then treated either with the freshly prepared water extracts or with different concentrations of lyophilized extract (0.2 mg/mL to 0.6 mg/mL. 

The G361 human melanoma cells were also treated with metformin (1,1-dimethylbiguanide hydrochloride- Aldrich, USA) at a concentration of 4 mM. This concentration was based upon work done by previous groups, which had used concentrations ranging from 0.5 to 10 mM metformin to treat human breast carcinoma cells [15]; Isakovic et al. used concentrations upto to 8 mM to treat glioma cells [16], and concentrations of metformin reached as high as 20 mM to inhibit mTOR in human breast carcinoma [14].

### 2.3. Cell Viability

Cell viability was measured using a WST-1reagent (Roche Applied Science, IN, USA). A375s G361s and NHFs were plated with a fixed number of cells per well 96-well tissue culture plates. After 24 hours, the cells were treated with DRE at the indicated concentrations. WST-1 reagent was added to the wells after the treatment period and incubated at 37°C for 4 hours. The plates were then read at 450 nm on a Wallac Victor^3^ 1420 Multilabel Counter (Perkin Elmer, ON, Canada). Absorbance readings are expressed in terms of cell viability as percent control (untreated cells).

### 2.4. Cellular Staining

Cells treated with DRE for the specified time periods were stained with Hoechst 33342 dye (final concentration 10 *μ*M) as per a previously published protocol [17], to image the cellular morphological features. Apoptotic cells are characterized by condensed, brightly stained nuclei. Nonapoptotic cells are not condensed or brightly stained. Cells were imaged with the dye using a fluorescent microscope (Leica DM IRB); pictures were taken at 40X objective.

### 2.5. Annexin-V Binding Assay

To confirm DRE-induced apoptosis in, Annexin-V binding assay was performed after 48 hours with DRE using a kit and manufacturer's protocol (Sigma-Aldrich, Oakville, ON, Canada). Posttreatment cells were washed twice with PBS and resuspended in Annexin-V binding buffer (10 mM HEPES, 10 mM NaOH, 140 mM NaCl, 1 mM CaCl_2_, pH 7.6). Annexin-V-FITC conjugate (1 : 50) was added to the cells and incubated for 15 minutes at room temperature. Cells were observed and imaged under a fluorescent microscope (Leica DM IRB); images were taken at 40x objective.

### 2.6. Caspase-8 Activity Assay

Caspase activity was assayed using a previously published protocol and manufacturer's procedure (Enzyme System Products, USA). Cell lysates were prepared using A375 human melanoma cells and incubated for 1 hour with caspase-8 fluorogenic substrate (IETD-AFC) in IETD buffer at 37°C (Calbiochem) in 96-well opaque microtiter plates. AFC fluorescence was measured at 400 nm excitation and 505 nm emission using spectrofluorometer (SpectraMax Gemini XS). Caspase activity was measured expressed in terms of per *μ*g protein, and protein concentration was determined using Bradford Assay (BioRad); Bovine Serum Albumin (BSA) was used as a standard.

### 2.7. Observation of Mitochondrial Membrane Potential Depolarization

Human melanoma A375 cells were seeded on sterile coverslips in 6-well tissue culture plates and treated with DRE for 24 hours. After DRE treatment, the cells were incubated for 1 hour with JC-1 dye (5,5′,6,6′-tetrachloro-1,1′,3,3′-tetraethyl-benzamidazolocarbocyanin iodide) at a final concentration of 0.5 *μ*M at 37°C. Cells were observed and imaged using a fluorescent microscope (Leica DM IRB); images were taken at 40x objective.

### 2.8. Measurement of Mitochondrial Reactive Oxygen Species

Mitochondria were isolated from A375 cells based on a previously published protocol [18]. The mitochondria were treated directly with DRE in 96-well opaque microtiter plates. Amplex Red (5 *μ*M) and horseradish peroxidase (HRP) were added to the mitochondria posttreatment. The fluorescent resorufin product was measured at 530 nm excitation and 580 nm emission using spectrofluorometer (SpectraMax Gemini XS). Relative Fluorescence Units were measured and expressed in terms of per *μ*g protein, and protein concentration was determined using Bradford Assay (BioRad); Bovine Serum Albumin (BSA) was used as a standard.

### 2.9. Immunoblotting

Whole cell lysates were prepared from treated cells and were run on 12% SDS-polyacrylamide gels. The gels were then transferred to a nitrocellulose membrane and probed for the proteins of interest (Bcl-2 and *β*-Actin- Santa Cruz Biotechnology). The blots were then probed with a secondary antibody conjugated to HRP and incubated, following which they were momentarily incubated with the chemiluminscent substrate (Sigma), before being exposed.

### 2.10. Posttreatment Cell Revival

A375 cells pretreated with DRE for 72 hours were trypsinized and replated in drug-free RPMI1640 media and incubated for 96 hours at 37°C and 5% CO_2_. Cells were stained using Hoechst 33342 dye and imaged using a fluorescent microscope (Leica DM IRB); pictures were taken at 40x objective. Cells were also collected and counted using Trypan blue exclusion assay.

### 2.11. High-Performance Liquid Chromatography

A high-performance liquid chromatography (HPLC) system, obtained from Waters Corporation (Mississauga, ON.), was used for separation and comparative analysis of Dandelion Root Extract (commercially available compared to that prepared by the Pandey Lab). The system consisted of a binary HPLC pump, an autosampler, a dual-wavelength absorbance detector, and a BDS HYPERSIL C18 reverse phase column (5 *μ*M, 150 × 4.6 mm) obtained from Thermo Electron Corporation (Palm Beach, FL), controlled by Breeze Software. Elutions were isocratic with a mobile phase consisting of methanol and 0.1% acetic acid (80 : 20 at 230 nm). The injection volume was 10 *μ*L with a flow rate of 1.0 mL/min. Each sample was run for three hours in triplicate.

## 3. Results

### 3.1. Effect of Dandelion Root Extract (DRE) on Human Melanoma Cell Viability

**Figure 1:**
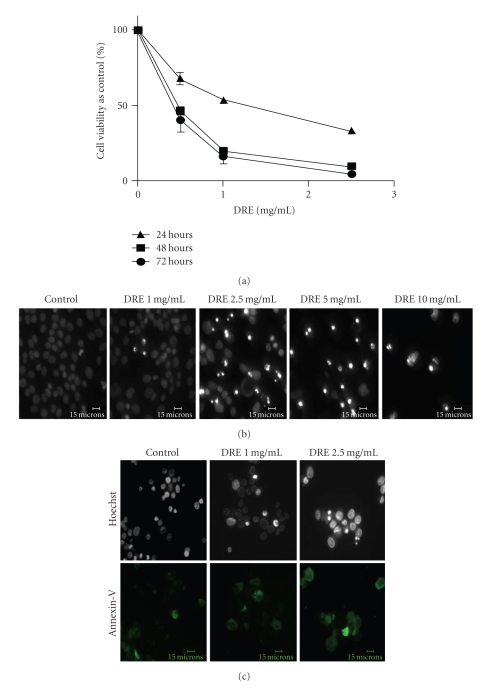
Determining the sensitivity of A375 human melanoma cells to DRE (a) Effect of DRE on cell viability. A375 human melanoma cells were seeded on 96-well plates (~1000 cells/well) and treated at the indicated concentrations for 24, 48, and 72 hours. The WST-1 dye was added to each well after every treatment period and incubated, as described in Section 2. Absorbances were read at 450 nm. (b) Induction of apoptosis by DRE. Typical apoptotic morphology was observed in A375 cells treated with DRE (0–10 mg/mL concentrations) for 48 hours. Cells were stained with Hoechst 33342 dye, before images were taken on a fluorescence microscope. Brightly stained, condensed bodies indicate apoptotic nuclei. (c) Confirmation of apoptosis by Annexin-V binding assay. Cells treated at the effective and subeffective doses of 2.5 and 1 mg/mL, respectively, for 48 hours were stained with Annexin-V Alexa Fluor (green) following which cells were imaged on a fluorescence microscope.

In order to investigate whether Dandelion Root Extract (DRE) reduced cell viability in human melanoma cells, the A375 cells were treated with 1, 2.5, and 5 mg/mL concentrations of DRE. DRE was found to reduce cell viability in a dose-dependent fashion, over time, in A375 melanoma cells as was measured by WST-1 assay. Based on metabolic activity of A375s, it was confirmed that treatment at 2.5 mg/mL DRE resulted in ~50% reduction in cell viability against control within 24 hours (Figure 1(a)). After cells were imaged with Hoechst dye, it was found that by 48 hours there was a clear induction of apoptosis at concentrations above 2.5 mg/mL (Figure 1(b)), as distinguished by brightly stained nuclei and the morphological features of condensation and fragmentation. 

Using the effective and subeffective doses, we sought to confirm that apoptosis in A375 cells was indeed induced, using the Annexin-V binding assay. The assay confirms that by 48 hours the phosphatidyl serine has flipped from the inner leaflet of the plasma membrane to the outer leaflet after treatment with 2.5 mg/mL DRE (Figure 1(c)).

### 3.2. Evaluation of DRE Toxicity on Normal Human Fibroblasts

**Figure 2:**
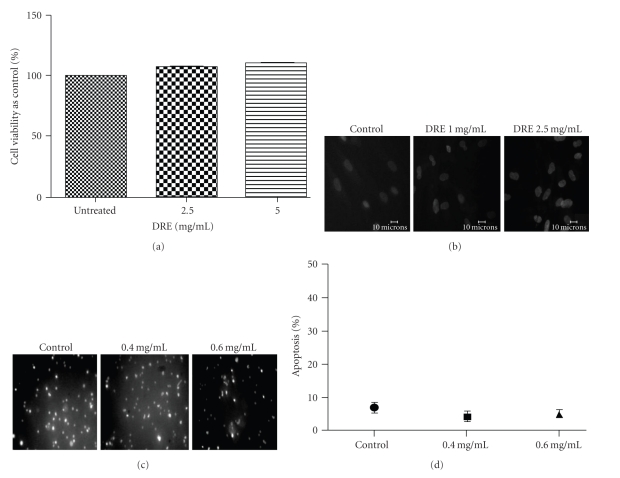
Determining the toxicity of DRE on Normal Human Fibroblasts (NHFs) (a) Effect of DRE on NHF cell viability. NHFs were seeded on a 96-well plate (~2000 cells/well) and treated with DRE at the indicated concentrations for 96 hours. The WST-1 dye was added to each well after the 96-hour treatment period and incubated as described in Section 2. Absorbances were read at 450 nm. (b) Nontoxic effect of DRE on NHFs. NHFs were treated with DRE at the indicated concentrations for 96 hours. Cells were stained with Hoechst 33342 dye and imaged ona fluorescence microscope, as described in Section 2 (c) Effect of DRE on peripheral mononuclear blood cells (PBMCs). PBMCs were treated at the indicated concentrations for 48 hours before being stained with Hoechst 33342 dye and imaged on a fluorescence microscope. (d) Nontoxic effect of DRE on PBMCs. PBMCs treated in Figure 2(c) were quantified by manual counting of brightly stained apoptotic nuclei.

With DRE having proven its efficacy in successfully killing this aggressive, chemoresistant form of skin cancer, DRE toxicity on normal cells had to be evaluated. Taraxacum extracts have been used for centuries and therefore it is assumed that normal cells are unaffected by it. Regardless, Normal Human Fibroblasts (NHFs) were used as a normal counterpart to human melanoma cells to test this assumption. DRE was treated at the effective and subeffective doses of DRE (1 and 2.5 mg/mL). After a long exposure of 96 hours, NHFs did not exhibit any reduction in cell viability (Figure 2(a)), and this was further supported by the lack of brightly stained, condensed and fragmented nuclei (characteristic of apoptosis) when stained with Hoechst dye (Figure 2(b)). Similarly, PBMCs (another normal cell line) treated with DRE were not susceptible to apoptosis induction after 48 hours of treatment (Figures 2(c) and 2(d)).

### 3.3. Analysis of Cell Death Pathways Employed by DRE

**Figure 3:**
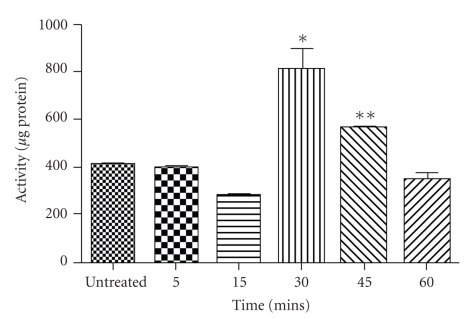
Caspase-8 activity in DRE-treated A375 cells. A375 cells were treated at 2.5 mg/mL for the indicated periods of time. Cells were collected and incubated with caspase-8 IETD-AFC substrate for an hour before being read on a spectrofluorometer as described in Section 2. Statistical analysis was performed using the GraphPad Prism 5.0; *denotes a *P*-value <.05; **denotes a *P*-value <.01.

To determine how apoptosis is being induced in A375 cells, we wanted to assess whether there was cell surface death receptor-mediated induction resulted in caspase-8 activation. Logically, we first chose a time point (24 hours) prior to when we had observed apoptosis (48 hours) by Hoechst staining with 2.5 mg/mL DRE (Figures 1(a) and 1(b)). However, this proved to be too late and caspase-8 activation occurred, cleaving the IETD-AFC substrate, within 30 minutes of DRE treatment (Figure 3).

**Figure 4:**
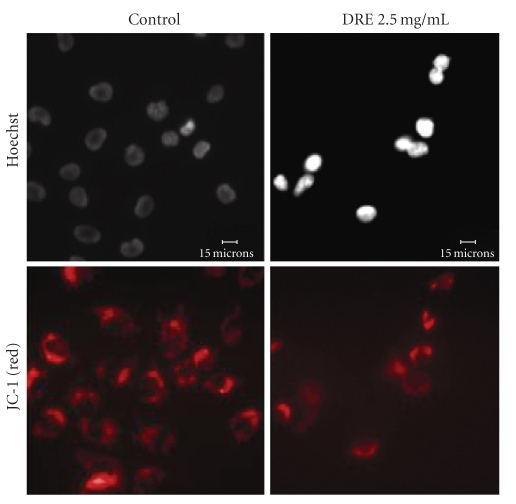
Dissipation of the mitochondrial membrane potential in A375 cells. A375 cells were seeded on coverslips and treated with DRE for 24 hours. The JC-1 was then added and cells were incubated for 1 hour, before being imaged on a fluorescent microscope, as described in Section 2. Punctate marks indicate healthy mitochondria.

Considering early activation of a death receptor-mediated pathway, we wanted to then assess the effect of DRE on the mitochondria by observing polarization/depolarization across the mitochondrial membrane using the JC-1 dye. The JC-1 dye moves into the mitochondria and will only aggregate if the mitochondrial membrane potential is maintained, as shown by red punctate marks. By 24 hours, the effective dose of 2.5 mg/mL DRE indicated the dissipation in the mitochondrial membrane potential, indicated by fewer punctate marks, as a result of the dye's inability to aggregate in the mitochondria with lost potential (Figure 4).

**Figure 5:**
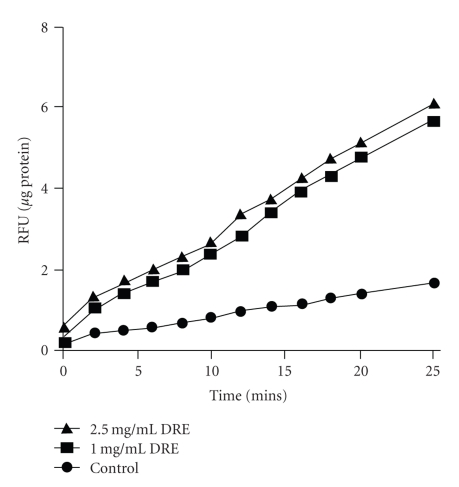
Assessing levels of DRE-induced ROS generation in A375 mitochondria. Mitochondria were isolated from A375 cells as described in Section 2.

 In order to determine whether the ROS generation in A375 melanoma cells was as a direct result of DRE or not, mitochondria isolated from the A375 melanoma cells [18] were treated with DRE at 1 and 2.5 mg/mL concentrations. Mitochondrial ROS was measured over 25 minutes for 2-minute intervals. A steady increase in the levels of mitochondrial ROS was observed compared to untreated control. At 0 minutes (*T* = 0), there is a 2.5 mg/mL DRE generated higher levels of reactive oxygen species than 1 mg/mL (data not shown). However, over time both concentrations showed the same rate of ROS production compared to control (Figure 5). 

**Figure 6:**
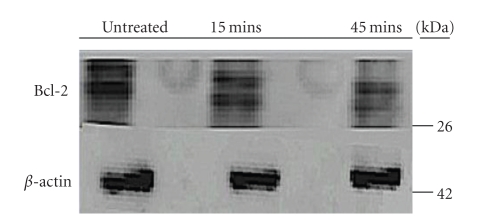
Reduction in Bcl-2 levels after treatment over time with DRE. A375 cells were treated with 2.5 mg/mL DRE for 15 and 45 minutes. Equal amounts of protein (20 *μ*g) were separate using SDS-PAGE. Western blotting with anti-Bcl-2 antibody assessed Bcl-2 levels. Equal protein loading was confirmed by *β*-actin immunoblotting.

Following this we observed the levels of Bcl-2 antiapoptotic protein from cells treated at 15 and 45 minutes. Bcl-2 levels are normally up-regulated in cancer cells as a means to safeguard them from apoptosis induction; this protein is also downstream of caspase-8. Though preliminary, we found that there was reduction in the levels of Bcl-2 protein early on at the 2.5 mg/mL dose, against the *β*-actin control (Figure 6).

### 3.4. Evaluating A375 Melanoma Cell Revival Posttreatment

**Figure 7:**
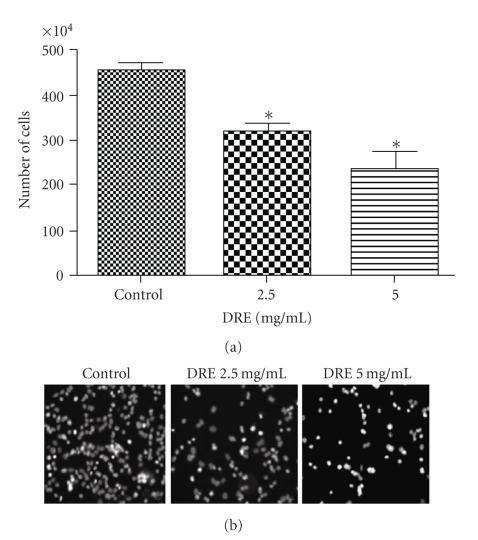
Determining A375 cell revival after DRE-treatment. (a) Negatively stained A375 cell count after DRE-treatment. A375 cells were treated with DRE, at the indicated concentrations, for 72 hours, following which they were replated in fresh drug-media and incubated for 96 hours as described in Section 2. Negatively stained cells were collected and counted using Trypan blue. Statistical analysis was performed using the GraphPad Prism 5.0; *denotes a *P*-value <.05. (b) Continued apoptosis induction in A375 cells after DRE-treatment. A375 cells were treated with DRE at the indicated concentrations for 72 hours, as described in Section 2 After 96 hours of incubation in drug-free media, cells were stained with Hoechst 33342 dye and imaged on a fluorescence microscope.

To ascertain the long-term efficacy of DRE on chemo-resistant melanoma, A375 cells were treated at 2.5 and 5 mg/mL for 72 hours before being replated in drug-free media and incubated for 96 hours. The Trypan blue count after 96 hours shows a reduction in cell viability upon increasing concentration. The viable cells counted also account for membrane unpermeabilized apoptotic cells (Figure 7(a)), and these apoptotic cells can be observed by Hoechst staining (Figure 7(b)). In (Figure 7(b)), the apoptotic nuclei can be clearly distinguished by the brightly stained nuclei.

### 3.5. Effect of DRE on G361 Human Melanoma Cells

**Figure 8:**
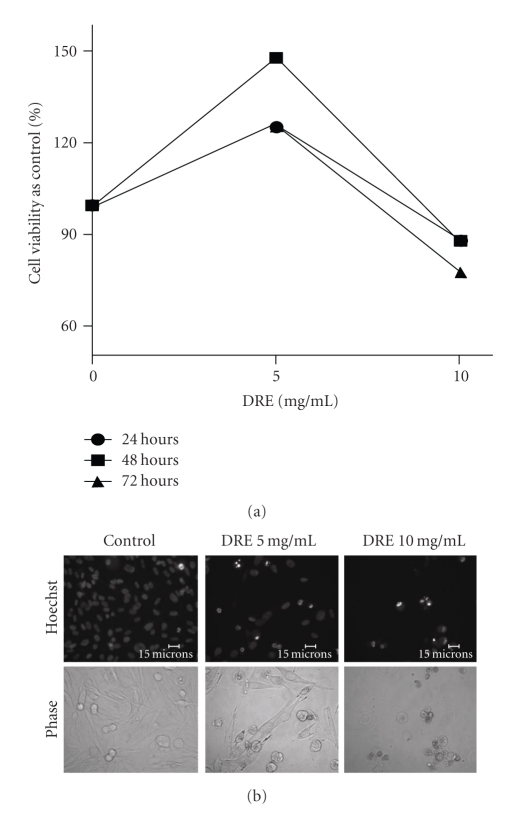
Effect of DRE on G361 human melanoma cells. (a) Effect of DRE on G361 cell viability. G361 human melanoma cells were seeded on 96-well plates (~3000 cells/well) and treated at the indicated concentrations for 72 hours. The WST-1 dye was added to each well after every treatment period and incubated, as described in Section 2. Absorbances were read at 450 nm. (b) Induction of apoptosis in G361 cells by DRE. Typical apoptotic morphology was observed in G361 cells treated with DRE at 10 mg/mL concentrations for 72 hours. Cells were stained with Hoechst 33342 dye, before images were taken on a fluorescence microscope. Brightly stained, condensed bodies indicate apoptotic nuclei.

**Figure 9:**
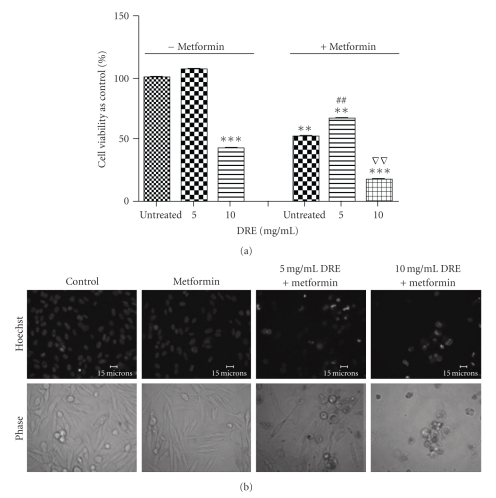
Combinatorial treatment of metformin and DRE on relatively resistant G361 cells. (a) Cell viability of relatively resistant G361 cells. G361 human melanoma cells were seeded on 96-well plates (~3000 cells/well) and treated at the indicated concentrations of DRE and 4 mM metformin for 72 hours. The WST-1 dye was added to each well after every treatment period and incubated, as described in Section 2. Absorbances were read at 450 nm. Statistical analysis was performed using the GraphPad Prism 5.0; **denotes a *P*-value <.05 compared to untreated control without metformin; ***denotes a *P*-value <.0005 compared to untreated control without metformin; ^##^denotes a *P*-value <.05 compared to 5 mg/mL DRE treatment without metformin; VV denotes a *P*-value <.05 compared to 10 mg/mL treatment without metformin. (b) Induction of apoptosis in metformin-treated G361 cells by DRE. Typical apoptotic morphology was observed in G361 cells treated with DRE starting at 5 mg/mL concentrations for 72 hours. Cells were stained with Hoechst 33342 dye, before images were taken on a fluorescence microscope. Brightly stained, condensed bodies indicate apoptotic nuclei.

To further determine the effect of DRE on other melanomas, we used the G361 human melanoma cell line and treated these cells for 72 hours at the same concentrations of DRE as the A375s. These doses proved to be ineffective in reducing cell viability and inducing apoptosis in G361 cells (data not shown). Higher doses were then used and a response was observed at a concentration of 10 mg/mL (Figures 8(a) and 8(b)). In combination with the anti-type II diabetes drug and metabolism interfering drug, metformin, these relatively more resistant melanoma cells were sensitized to the effects of DRE even at lower doses of 5 mg/mL (Figures 9(a) and 9(b)).

## 4. Discussion

Dandelion Root Extract (DRE) has thus far been used in traditional medicine as a detoxifying agent for digestive disorders, for lung, breast, and uterine tumours [2], and most interestingly, to treat chronic diseases of the skin [4]. However, there has been little scientific advancement made in this field with regard to the effect of dandelion root extract on cancer, and even more so on chemoresistant, human malignant melanoma skin cancer. Previous work with Taraxacum has not provided much mechanistic detail with regards to apoptosis induction, instead highlighting its antioxidant and anti-inflammatory effects. In this study of human melanoma cells, we show that Dandelion Root Extract (DRE) is more than a worthy chemopreventative, it is fast-acting, nontoxic, and therefore specific in its targeting of human melanoma cancer cells, making it a valuable chemotherapeutic. We have investigated the induction of apoptosis in human malignant melanoma cells and observed its long-term effects in human melanoma cancer. 

The WST-1 assay (the readings of which are returned as a function of metabolic activity of mitochondrial dehydrogenases) reported reductions in A375 cell viability in a time- and dose-dependent manner upon DRE treatment (Figure 1(a)). By 48 hours, human melanoma A375 cells uncharacteristically showed susceptibility to apoptosis induction by DRE (Figure 1(b)), displaying morphological features of condensed and fragmented nuclei—typical of apoptotic cells. Based on cell viability and observation of extent of apoptosis by 48 hours, we established the effective dose as 2.5 mg/mL. Given that DRE has traditionally been used naturopathically for a variety of ailments, we assume that it would be relatively nontoxic to healthy cells. Our results show that the Normal Human Fibroblasts (NHFs) (which were treated at a low population doubling where NHFs have the best proliferation rate) and Peripheral Blood Mononuclear Cells remained unaffected and healthy after a 96-hour and 48- hour exposure to DRE, respectively (Figures 2(a)–2(d)).

 Dandelion root extract has been resolved into components using chromatography techniques [6]; however, singular components themselves may not be enough to trigger a chemotherapeutic response in a chemoresistant cancer. Components may require each other to work in unison or even synergy, which is possibly why they have been effective as extracts in traditional medicines. With one of the triterpene components of DRE, Lupeol, Hata et al. observed a decrease in mouse melanoma differentiation [7]. This study has been supported by a two-stage skin carcinogenesis mouse model showing antiproliferative and chemopreventative activity of this compound [19]. However, initial *in vivo *studies with *Taraxacum japonicum*, conducted by Takasaki et al., concluded that it was the taraxasterol component that was the worthy cancer chemopreventative [9]. Though the extract is constituted of a myriad of compounds, we wanted to determine the resultant mechanistic effect of the combined components in specifically killing human melanoma cells. 

Caspase-8 activation results from the binding of ligands (such as Fas) to the death receptors (such as Fas receptors) on the cell surface [20]. Fas-receptor-mediated apoptosis would result in recruitment and conversion of pro-caspase-8 to active caspase-8. In this study, we observed the rapid activation of caspase-8 enzyme in A375 cells, and this is corroborated by a previous report by Ariza et al. stating that these cells do express the Fas receptor, thus strengthening our theory that an extrinsic apoptotic pathway is activated [21].

Judging by the diminished activity of the mitochondrial dehydrogenases (Figure 1(a)), we also visualized dissipation of the mitochondrial membrane potential at 24 hours of treatment using JC-1. This indicates that the mitochondria is depolarized early on, and this in turn agrees with the induction of apoptosis that we observe at 48 hours of treatment, following the dissipation. Direct mitochondrial destabilization also occurred upon DRE treatment indicating that DRE action is not only cell death receptor-mediated, but that its effect on the mitochondria may not be purely resultant, but a consequence of direct mitochondria targeting and even possible cross talking between the extrinsic and intrinsic pathways. More importantly, an increase in ROS production indicates prooxidant behaviour of DRE on cancer cell mitochondria, which is contrary to the antioxidant convictions of traditional medicine and previous studies on Taraxacum extracts citing reductions in NO, ROS, RNS, and COX-2 [10, 11] in mouse macrophages. This duality in Taraxacum's operation may depend on the cell's nature—normal versus cancerous—further underlining Taraxacum's ability to distinguish between these cells.

With early activation of caspase-8 induction, we had also observed, in a preliminary study, a decrease in the levels of Bcl-2 protein within 45 minutes compared to control (Figure 6). In normal cells, keratinocytes, which regulate melanocytes, promote Bcl-2 expression [22]. Though the role of Bcl-2 expression in melanoma remains controversial in terms of tumourigenesis initiation, the cells are sure to exploit the high levels of endogenous Bcl-2 to survive [1]. Previous studies have already shown that antisense silencing of Bcl-2 sabotages melanoma survival, facilitating effective melanoma chemotherapy [23].

There are two main points that must be stated here: firstly, that noncancerous cells are unaffected by DRE treatment, and secondly, melanoma cells retain the signals to commit suicide long after DRE has been removed from the system. These cells correspond to those which remain nonapoptotic after the initial 96-hour treatment with DRE. The cell count in Figure 7(a) represents negative Trypan blue staining which indicates membrane unpermeabilized cells-viable and also apoptotic cells. Hoechst images corroborate the Trypan counts by revealing brightly stained nuclei after 96 hours mostly at 5 mg/mL DRE. These cells, therefore, have retained the signals to commit suicide long after the drug has been removed, making it a worthy chemotherapeutic (Figure 7(b)).

 Upon comparison of A375 melanoma with G361, we found that the latter did not respond to the same doses and only started to respond, though minimally, to DRE at much higher concentrations (10 mg/mL)—about four times the effective concentration for A375 (Figures 8(a) and 8(b)). Since WST-1 assay showed increased susceptibility of only A375 to DRE (Figure 1(a)), measured at the mitochondrial level, differences between these cell lines could highlight the mechanism by which DRE is acting to induce programmed cell death in one cell line (A375) while being resisted by the second (G361). According to Su et al., in a microarray study comparing different melanoma cell lines for gene expression, it was found that G361 and A375s have varying levels of antioxidant and anti- and pro-apoptotic genes that are expressed [24]. For example, at basal conditions, the antioxidant, prosurvival gene, ATOX1, is up-regulated, and the pro-apoptotic gene, CASP4, is down-regulated in G361, but is uninduced in A375 in both cases. Quantification of gene expression using qRT-PCR, by Su et al., showed significant up-regulation in 3 antiapoptotic genes (PHB, PPP2R1B, and OPA) and the antioxidant gene for glutathione reductase (GSR). We could speculate that a combination of these factors could contribute to why G361 does not respond to DRE treatment, and by eliminating these factors, we could possibly determine how DRE might therefore act in A375 human melanoma cells.

With the relatively resistant G361 cells not responding to DRE treatment, we used the anti-type-II diabetes drug, metformin, to sensitize the cells. The energy metabolism of cancer cells being different from normal cells potentiates this difference as a point of specific vulnerability [14]. Metformin acts as a metabolism interfering compound that debilitates cancer cells, and the case of G361-resistant melanoma cells, combining DRE with metformin reduces cell viability at even lower doses (Figures 9(a) and 9(b)). Up to date work on human melanoma cells treated with metformin has been unprecedented.  Figure 9(a) shows that cell viability is reduced with metformin treatment alone. However, morphologically, under the microscope, we found that the number of cells was unaffected by metformin treatment alone (1 mM–8 mM). This means cell viability reduction was due to metformin inhibiting some of the metabolic enzymes in these cells, but without affecting the cell number (data not shown). In all other cases, there was always a correlation with the WST-1 activity and viability/cell number. 

 The dandelions that we had collected for this investigation were harvested in the month of May. Previous studies with Canadian dandelion have stated that during Autumn senescence, there is a accumulation of amino acids in the roots, but the levels of amino acids diminish in Spring, resulting in fluctuations between asparagine and glutamine across the seasons [25]. This is indicative that our extract is not primarily amino acid based. 

As mentioned before, various components like Lupeol have been considered as chemopreventatives for cancer. Components of DRE include sesquiterpenes (derivatives of germacranolide, eudesmanolide, and guaianolide), different triterpenes like taraxasterol—their hydroxy derivatives and their acetates—phenolics compounds (such as chicoric acid, vanillic acid, p-hydroxyphenylacetic acid, p-hydroxybenzoic acid, syringic acid, caffeic acid, chlorogenic acid, and ferulic acid), and coumarins (scopoletin, esculetin, and umbelliferone) [26]. We are yet to determine the effect of each of the individual components (such as the family of triterpene alcohols and phenolic acids—found in the roots—and cinnamic acids, flavinoids and coumarins—that are found in the leaves) [27] but we believe that the compounds in DRE most likely work in synergy with each other to produce the aforementioned resultant effect. We have performed high performance liquid chromatography (HPLC) with our water soluble DRE and compared it to a commercially available DRE. Both extracts produced similar profiles (data not shown). We have previously performed protease digestion on DRE and found that it did not have any effect on its activity on cancerous cells. In this study, we are narrowing on the therapeutic potential of dandelion root extract rather than its caliber as a prophylactic agent. We believe that this nontoxic extract can undergo precipitous translation from bench top to bedside, with dandelion products that are already commercially available in the form of tea and supplements. Traditional therapeutics have provided us a new scope for harnessing the potential of natural extracts in modern medicine; the efficacy of DRE is only fully being realized now as a chemotherapeutic against aggressive chemoresistant cancers.
